# Serotonin 1B receptor density mapping of the human brainstem using positron emission tomography and autoradiography

**DOI:** 10.1177/0271678X211049185

**Published:** 2021-10-13

**Authors:** Emma R Veldman, Andrea Varrone, Katarina Varnäs, Marie M Svedberg, Zsolt Cselényi, Mikael Tiger, Balázs Gulyás, Christer Halldin, Johan Lundberg

**Affiliations:** 1 Department of Clinical Neuroscience, Center for Psychiatry Research, Karolinska Institutet and Stockholm County Council, Stockholm, Sweden; 2 Department of Health Promotion Science, Sophiahemmet University, Stockholm, Sweden; 3 PET Science Centre, Personalized Medicine and Biosamples, R&D, AstraZeneca, Stockholm, Sweden; 4 Lee Kong Chian School of Medicine, Nanyang Technological University, Singapore, Singapore

**Keywords:** Serotonin 1B (5-HT1B), positron emission tomography (PET), human, 3D autoradiography, brainstem

## Abstract

The serotonin 1B (5-HT_1B_) receptor has lately received considerable interest in relation to psychiatric and neurological diseases, partly due to findings based on quantification using Positron Emission Tomography (PET). Although the brainstem is an important structure in this regard, PET radioligand binding quantification in brainstem areas often shows poor reliability. This study aims to improve PET quantification of 5-HT_1B_ receptor binding in the brainstem.

Volumes of interest (VOIs) were selected based on a 3D [^3^H]AZ10419369 Autoradiography brainstem model, which visualized 5-HT_1B_ receptor distribution in high resolution. Two previously developed VOI delineation methods were tested and compared to a conventional manual method. For a method based on template data, a [^11^C]AZ10419369 PET template was created by averaging parametric binding potential (BP_ND_) images of 52 healthy subjects. VOIs were generated based on a predefined volume and BP_ND_ thresholding and subsequently applied to test-retest [^11^C]AZ10419369 parametric BP_ND_ images of 8 healthy subjects. For a method based on individual subject data, VOIs were generated directly on each individual parametric image.

Both methods showed improved reliability compared to a conventional manual VOI. The VOIs created with [^11^C]AZ10419369 template data can be automatically applied to future PET studies measuring 5-HT_1B_ receptor binding in the brainstem.

## Introduction

The brainstem contains various nuclei, from which, among others, mono-aminergic projections originate. For this reason, neuroreceptor mapping of brainstem with *in vivo* Positron Emission Tomography (PET) is key to study the pathophysiology and treatment of psychiatric and neurological disorders. For delineation of Volumes of Interest (VOIs) on PET images, Magnetic Resonance Imaging (MRI) is conventionally used as structural reference. In the case of brainstem nuclei, however, the anatomical localization is challenged by their small size and poor visibility with standard MRI sequences. In addition, their visualization on PET images can be affected by partial volume effects (PVE), due to spill-in, i.e. the contribution of radioactivity from nearby regions, and spill-out, i.e. the loss of radioactivity from the target region.^1^ These limitations can be avoided using *post mortem* autoradiography (ARG). ARG uses radioisotopes with much lower energy (e.g ^3^H, energy: 6 keV) than PET radioisotopes (e.g. ^11^C, energy: 967 keV).^2^ Moreover, the ARG signal is detected on close proximity to the tissue slice, whereas resolution in PET is more affected by attenuation occurring in tissue and scatter in the larger distance between object and detector. Therefore, ARG enables to map and quantify brain targets with much higher resolution (<0.1 mm),^3^ in comparison to PET, even in the case of using the high-resolution research tomograph (HRRT) with a resolution of 2–3 mm.^4^ In small regions such as the brainstem nuclei, ARG provides reliable measures of protein density with high anatomical resolution and can therefore complement protein density quantification with PET.

The serotonin 1B (5-HT_1B_) receptor is a target of interest in research of the pathophysiology and treatment of major depressive disorder (MDD).^5,6^ In MDD patients, lower 5-HT_1B_ receptor availability has been found in hippocampus, anterior cingulate cortex^7,8^ and a volume containing the ventral striatum and pallidum.^9^ Increased 5-HT_1B_ receptor availability in cortex has been shown after a single administration of the antidepressant escitalopram.^10^ Moreover, single ketamine treatment showed an increase in 5-HT_1B_ receptor binding in the hippocampus, although not significant when compared to a placebo.^5^ Within the brainstem however, results seem less clear: animal models of depression were shown to have increased 5-HT_1B_ receptor expression in the dorsal raphe nuclei,^11^ while overexpression of the 5-HT_1B_ receptor in caudal dorsal raphe nuclei showed reduced depression-like symptoms.^12^ Human PET studies have previously shown reduced 5-HT_1B_ receptor binding in a dorsal region of the brainstem after administration of a single dose of escitalopram^10^ and after treatment of patients with major depression with cognitive behavior therapy.^13^ However, test-retest data showed high variability of 5-HT_1B_ receptor density quantification in this area.^14^ Therefore, methods to improve 5-HT_1B_ receptor density quantifications in brainstem are highly desired. Processing methods such as those to define VOIs have a large impact on outcome values.^15^

Methods for VOI definition which improve protein density quantification in brainstem nuclei have so far mainly been focusing on determining serotonin transporter (5-HTT) and 5-HT_1A_ density.^16–18^ Whether these methods are applicable for 5-HT_1B_ receptor density quantification is uncertain, as the 5-HT_1B_ receptor has been shown *in vitro* to possess a different distribution pattern and receptor density.^19^ While in substantia nigra and the central gray area specifically high 5-HT_1B_ receptor densities were found,^20,21^ the 5-HT_1A_ receptor and 5-HTT were found to be more abundant in the dorsal raphe nuclei and superior colliculi.^19,22^ More caudally located, 5-HTT’s are present in the raphe magnus and raphe obscurus, while 5-HT_1B_ receptors were shown in the solitary nucleus, the trigeminal nerve nucleus pars caudalis and the substantia gelatinosa.^23^ In rodents, substantial 5-HT_1B_ receptor densities have also been found in the pons.^24^ This difference in localization is likely to be explained by their functional differences: while the 5-HT1A receptor and 5-HTT is only located on serotonergic neurons, 5-HT_1B_ receptors can also function as heteroreceptors on other neuron types.^25^ Therefore, delineation methods to determine 5-HT_1A_ receptor or 5-HTT densities in the brainstem might not be suitable for the purpose of 5-HT_1B_ receptor quantification.

The aim of the current study was to improve the PET quantification of 5-HT_1B_ receptor densities in the brainstem by studying high-resolution receptor distributions using ARG and [^3^H]AZ10419369, a selective 5-HT_1B_ receptor ligand.^26^ Appropriate VOIs for PET quantification were defined and delineation methods were tested in order to develop an adequate quantification method of the 5-HT_1B_ receptor ligand with [^11^C]AZ10419369 in the brainstem.

## Material and methods

The distribution of the 5-HT_1B_ receptor was studied *post mortem* in brainstem tissue at high spatial resolution by creating a 3D model of [^3^H]AZ10419369 autoradiograms. This 3D ARG model was used to guide brainstem VOI selection for [^11^C]AZ10419369 PET image analysis, in which two different VOI-defining methods were tested and compared for reliability. The work in this study, and studies of which data used in this study originated, were performed in accordance to the Declaration of Helsinki.

### Autoradiography

#### Materials

[^3^H]AZ10419369 (molar activity 83 Ci/mmol), radiochemical purity >98%, was purchased from Novandi Chemistry AB (Södertälje, Sweden). All other chemicals were obtained from commercially available sources and were of analytical grade.

#### Human postmortem brain tissue

Human postmortem brain tissue was obtained with family consent at the Department of Forensic and Insurance Medicine, Semmelweis Medical University (Budapest, Hungary). Studies were approved by the Ethics Committee of Karolinska Institutet (Dnr. 03-767) and the Semmelweis University Human Ethical Committee (Dnr. 113/1995, 180/2001). Brain tissue from two donors was used, of which one was used for the final 3D visualization of brainstem 5-HT_1B_ receptors. None of the donors had known history or symptoms of neurological or psychiatric disorders and none of the brains exhibited damages or abnormalities from examination at autopsy and during sectioning. See Table 1 for specifications.

**Table 1.** Specifications of postmortem brain tissue.

Brain no.	Anatomical plane	Age (y)	Gender	PMI (h)	Cause of death
1	Sagittal (right hemisphere)	68	M	<24	Right ventricle failure
2	Axial	34	F	<24	Thrombotic occlusion of pulmonary arteries

PMI: post mortem interval.

Brainstem tissue was sectioned into 20 µm-thick sagittal or axial sections using a cryomicrotome (Leica CM 1860). Sections were thaw-mounted to poly-l-lysine-treated glass plates (size 7.5 × 3.5 cm) and stored frozen at −20°C until use. Sagittal sections from the right hemisphere, used for visualization of total binding in the autoradiographic procedure, were chosen leaving 0.5 mm of tissue between each consecutive slide. Photographic images were digitally recorded of the frozen tissue before cutting of each section and at the end of the cryostat wheel crank course, as previously described by Dubois et al., 2010.^27^

#### [^3^H]AZ10419369 in vitro autoradiography

The ARG experiments were performed essentially as described previously,^28^ with the addition of 0.1% BSA to the incubation buffer to decrease non-specific binding, e.g. to the tissue embedding material carboxymethyl cellulose. Sections used for autoradiography were subsequently used for Nissl staining. See supplementary materials 1.1 for a brief summary.

#### Volume rendering of ARG brainstem slices

Image preprocessing was performed using Fiji/ImageJ,^29^ version 1.50i. Photographic images taken during cryosectioning were used as a reference volume for the processed ARG and Nissl-stained brainstem sections, to control for possible sectioning related deformations.^30^ These photographic images were aligned and stacked using the Trakem2 plug-in.^31^ The Nissl-stained slices were then aligned and co-registered section-to-section to the photographic volume using the BUnwarpJ plug-in.^32^ A stack of ARG binding slices was created by aligning these to the Nissl-stained slices and co-registered using the matrix of the photographic images. Images representing non-specific binding were warped to the corresponding image with total binding. Subsequently, an ARG specific binding stack was generated by subtracting the information of the non-specific binding slices from the total binding slices before mirroring of the stack to represent ARG binding in a complete brainstem.

The ARG brainstem volume was transformed into the standard reference space of the Montréal Neurological Institute (MNI)^33^ as follows: first, the ARG volume was resized and resliced into the volume with the dimensions of the MNI-space. A rough repositioning on the brainstem of a standard MNI-brain was then performed manually upon visual inspection, using SPM12 (Wellcome Department of Cognitive Neurology, University College London). To get a more refined co-registration, co-localization information from a high signal region in the ARG stack and a clearly visible region in MNI-brain was necessary. Highest ARG signal was found in the substantia nigra, confirmed by Nissl-staining. Therefore, a substantia nigra-weighted brainstem of a standard MNI-brain was created, using the ATAG template.^34^ The ARG volume was then co-registered using a rigid-body and scaling (9-parameter) affine transformation to the adjusted MNI brainstem, using SPM12. To further match to the brainstem in MNI-space, a non-linear, warping registration was performed using the ‘Nifty_reg’ package.^35^ ARG images of [^3^H]AZ10419369 on axial slices were used to confirm binding localization visually in the axial plane.

**Figure 1.**
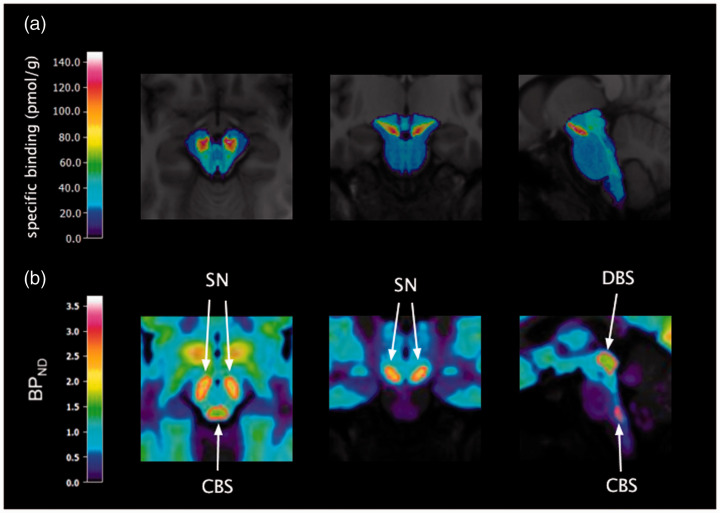
(a) [^3^H]AZ10419369 ARG binding in the brainstem transformed to MNI space, overlaid on the template MR image; (b) template VOIs (in red) of the dorsal brainstem (DBS), substantia nigra (SN) and caudal brainstem (CBS) on the template [^11^C]AZ10419369 PET image.

The pattern of distribution of specific binding in the brainstem can be seen in Figure 1(a). Furthermore, the full 3D ARG model was visualized using volume rendering with the software ParaView.^36^ More specifically, the isosurfaces at selected, distinct specific binding levels were rendered as semitransparent “glass shells” with color dependent on signal intensity essentially following the “jet” color map in MATLAB (R2019a, version 9.6. Natick, Massachusetts: The Mathworks Inc.). The video can be accessed as an online attachment (Video 1).

### Positron emission tomography

#### Participants

The current study included data from 60 healthy subjects, retrieved between 2010 and 2017, of which 52 were pooled and used to create a database. This includes data from 33 subjects of which results have been previously published.^10,37–39^ Furthermore, 8 subjects were part of a test-retest study^14^ and were in this study used for testing purposes. The database consisted of 32 male and 20 female subjects, 38.2 ± 17.9 years of age (mean ± SD); the testing subjects were all male and 23.0 ± 2.3 years of age.

Health of the subjects was confirmed by medical history and physical examination, including routine blood tests and MRI of the brain. All subjects gave written informed consent before participating in the study. The studies were approved by the Ethics Committee of the Stockholm region (Dnrs 2009/304-31, 2009/1021, 2010/089, 2010/672-31/3, 2011/791-31/4, 2014/452-31) and by the Radiation Safety Committee of the Karolinska University Hospital, Solna, Sweden.

#### PET measurements

Test-retest subjects underwent two PET examinations on the same day, remaining subjects were examined once. All PET examinations were performed using the same PET-system: the high-resolution research tomograph (HRRT, Siemens Molecular Imaging, Knoxville, USA). A bolus injection of [^11^C]AZ10419369 was administered with a radioactivity of 378 ± 54 MBq and 410 ± 15 MBq, for the database and the test-retest subjects respectively. The molar activity was respectively 375 ± 164 MBq and 441 ± 94 GBq/µmol and the injected mass was respectively 0.62 ± 0.51 and 0.45 ± 0.09 µg. Subsequently, the intravenous line was flushed with saline. To minimize head motion during the PET examination, an individually made plaster helmet was used.^40^ Emission data was acquired in list mode for 63 to 93 min and dynamic images were reconstructed in a series of 32 to 37 time frames using 3D ordinary Poisson ordered subset expectation maximization, including modeling of the system’s point spread function. This reconstruction protocol has previously been shown to provide a resolution of approximately 1.5 mm full width half maximum in the center of the field of view and 2.4 mm at 10 cm away from the center.^41^ To enable pooling of all PET data, the first 32 time frames were used (consisting of 8 × 10 s, 5 × 20 s, 4 × 30 s, 4 × 1 min, 4 × 3 min, 7 × 6 min).

#### Magnetic resonance imaging

T1-weighted MR images were acquired on a 3 T GE MR750 scanner (GE Medical Systems, Milwaukee, WI), except for two subjects in the template database, for which a 1.5 T GE Signa (GE Medical Systems, Milwaukee, WI) or 1.5 T Magnetom Avanto (Siemens Medical Solutions, PA, USA) system was used. The MR images were segmented into gray matter, white matter and cerebrospinal fluid using SPM12. Furthermore, the MR images were co-registered to the PET images to then transform VOIs from MRI to PET space.

#### PET image analysis

Images were corrected for minor head motion with a post-reconstruction frame-to-frame correction realignment algorithm, in which frames are realigned to the first four frames, as previously described by Schain et al., 2012.^42^ In the case of excessive head motion (e.g. >2 mm in z-direction), an additional motion correction algorithm based on the simplex method was applied.^43^ All PET data underwent minor motion correction using a frame-by-frame correction algorithm from SPM12. A wavelet-aided parametric imaging (WAPI) algorithm^44^ was used to create parametric BP_ND_ images. This approach uses wavelet-based denoising and employs the non-invasive, multilinear variant of Logan’s graphical analysis (here using the last 13 frames, t* = 23 min) to obtain BP_ND_ in each voxel in the brain. As previously described by Schain et al.,^16^ this method overcomes the VOI-size limitation of VOI-based compartment modeling and can therefore be used to quantify small brainstem regions.

#### Development of 5-HT_1B_ PET template

A [^11^C]AZ10419369 template was created with T1-weighted MR images and parametric PET images of 52 subjects. T1-weighted MR images were normalized to the standard reference MNI space^33^ using FSL (FSL 5.0, Oxford).^45^ After skull-stripping with BET,^46^ linear transformation was performed with FLIRT,^47^ followed by non-linear transformation with FNIRT.^48^ The inverse MRI-to-PET co-registration transform and the obtained non-linear transformation parameters were then used to normalize the parametric images into MNI-space. Subsequently, average images were created from both modalities.

A cerebellum VOI was automatically obtained as previously described by Matheson et al.^49^ This cerebellum VOI is restricted to a smaller region to avoid spill-over from the occipital cortex and cerebrospinal fluid. Furthermore this VOI avoids the cerebellar vermis, which has been shown to influence BP_ND_ significantly in neocortical VOIs measuring [^11^C]AZ10419369 binding.^50^

#### Definition of brainstem VOIs

The selection of suitable VOIs for quantification of 5-HT_1B_ receptor binding in the brainstem was based on 2D ARG data reported in the literature (see Introduction) and visual inspection of the 3D [^3^H]AZ10419369 ARG model. The homogeneity and continuity of 5-HT_1B_ receptor binding in each VOI, as well as the possibility to differentiate the specific signal from background and from other VOIs were considered in order to define the most appropriate regions. Three VOIs were thus selected: 1) a dorsal brainstem VOI, including dorsal and median raphe nuclei, periaqueductal gray, superior and inferior colliculi; 2) a substantia nigra VOI; and 3) a caudal brainstem VOI encompassing the 5-HT_1B_ receptors in the caudal part of the pons and medulla oblongata (see Introduction).

As a first step, for each VOI, a liberally sized volume was manually drawn in FSL on the previously acquired average MR images in standard MNI-space. Localization of these VOIs was guided by landmarks reported in literature.^51,52^ The final VOIs were generated and applied on test-retest data of 8 subjects by two previously reported methods based on: 1) individual PET data^16^ and 2) template PET data^17^ of [^11^C]AZ10419369 binding. Both methods were compared to results using the conventional method, i.e. a manually drawn VOI.

For the individually based method, the manually drawn initial volume was transformed into individual space, and then eroded to include the highest BP_ND_ within a fixed volume (see Table S1) based on findings in literature.^53–56^ To our knowledge, no reliable volumetric information has been reported on the caudal regions with known 5-HT_1B_ receptor densities. Moreover, visual inspection of the 3D ARG model showed a continuous, rather than discrete, distribution of 5-HT_1B_ receptor binding with relatively low density throughout the caudal part of the pons and medulla. Therefore, a caudal brainstem VOI was created in this area, by setting the threshold for the size to result in highest BP_ND_ values, while providing a continuous VOI mask with good reliability.^16^

Template based VOIs were instead eroded in the standard template space, which was acquired as described above. An estimation of non-linear normalization parameters was obtained for the test subjects in order to transform the template VOIs into individual space. Volumes of VOIs acquired with this method were therefore dependent on the brain structure sizes of each individual relatively to the template brain in MNI-space. Compared to the method performed by Fazio et al.,^17^ a less liberal threshold of 25% was used for the transformation to individual space, which improved the balance of VOI size and reliable outcome measures. The resulting VOIs were applied to each individual MR image to confirm consistency with individual brainstem anatomy. Subsequently, VOIs were transformed to PET space and applied on the individual parametric images to extract average BP_ND_ values for each VOI. See Figure 1(b) for the eroded VOIs in template space.

#### Comparison of PET and ARG data

Additionally, PET template binding data in the eroded VOIs was compared with 3D ARG signal in MNI-space to evaluate potential areas of discordance. This was executed by first normalizing BP_ND_ and specific binding values in each voxel to the highest binding value in the brainstem for PET and ARG data respectively. Subsequently, normalized PET BP_ND_ values were divided voxel-by-voxel by normalized specific binding values of the 3D ARG model. The data is corrected for age in the CBS and DBS VOI using linear regression, as in these regions a significant negative correlation with BP_ND_ values was seen (p < 0.001; r = −0.78 and r = −0.68 for CBS and DBS VOI, respectively). The distribution of voxels showing similar relative agreement between PET and ARG data was plotted.

### Statistical analysis

All statistical analyses were performed in R (version 3.6.3).^57^ Normal distribution of the data was assessed by visual inspection of density and QQ plots. To compare the test-retest outcomes for the two VOI-defining methods and assess their reliability, the following metrics were defined: Coefficient of Variation (COV), average Absolute Percentage Difference (APD), Intraclass Correlation Coefficient (ICC), Standard Error of Measurement (SEM) and Minimal Detectable difference (MD). The COV shows variation between subjects and is calculated by dividing the SD by the mean BP_ND_. The APD represents an absolute value of normalized intra-subject differences and is calculated as follows:

$$\mathrm{APD}=\frac{\left|{BP}_{ND}^{\mathrm{PET}2}-\;{BP}_{ND}^{\mathrm{PET}1}\right|}{\frac{1}{2}(\left|{BP}_{ND}^{\mathrm{PET}2}+{BP}_{ND}^{\mathrm{PET}1}\right|)}$$

The ICC is a measure of the inter-subject differentiability. In this case the one-way random effects model with single measures is used (i.e. ICC(1,1)). ICC values range from 0–1, with 1 indicating perfect inter-subject differentiability. The SEM represents an estimate of precision of individual outcome values and is given in the unit of the measurement (here: BP_ND_) and is calculated as follows:

$$\mathrm{SEM}=SD\;\sqrt{1-\mathrm{ICC}}$$

In which SD is based on all the PET measurements. Lastly, the MD gives the difference between two measurements necessary to be considered greater than random measurement error, according to a 95% confidence interval.^58^ Here, the MD is given as percentage of the average BP_ND_.

### Data and code availability

Resulting VOI masks based on [^11^C]AZ10419369 template PET template data of 52 subjects is available for download on: https://github.com/EmmaRV/3DARG_5HT1B. PET analyses were performed using in-house software. A full description of the 3D ARG volume rendering process, code for the created Fiji macro’s for this study and the ARG data in MNI-space can be found on the above mentioned webpage.

## Results

### Autoradiography in brainstem sections

The 3D-model created from brainstem sections with [^3^H]AZ10419369 ARG binding, after subtraction of non-specific binding, is seen in Video 1 and the brainstem ARG binding in MNI-space is seen in Figure 1(a). The 3D ARG model showed continuous distribution of [^3^H]AZ10419369 binding in the dorsal midbrain and from the caudal area of the pons to the medulla oblongata. Highest binding densities were seen in the substantia nigra. Regional specific binding values using conventional ARG delineation of ROIs can be found in Table S3.

### PET VOI-delineation methods in brainstem

**Figure 2.**
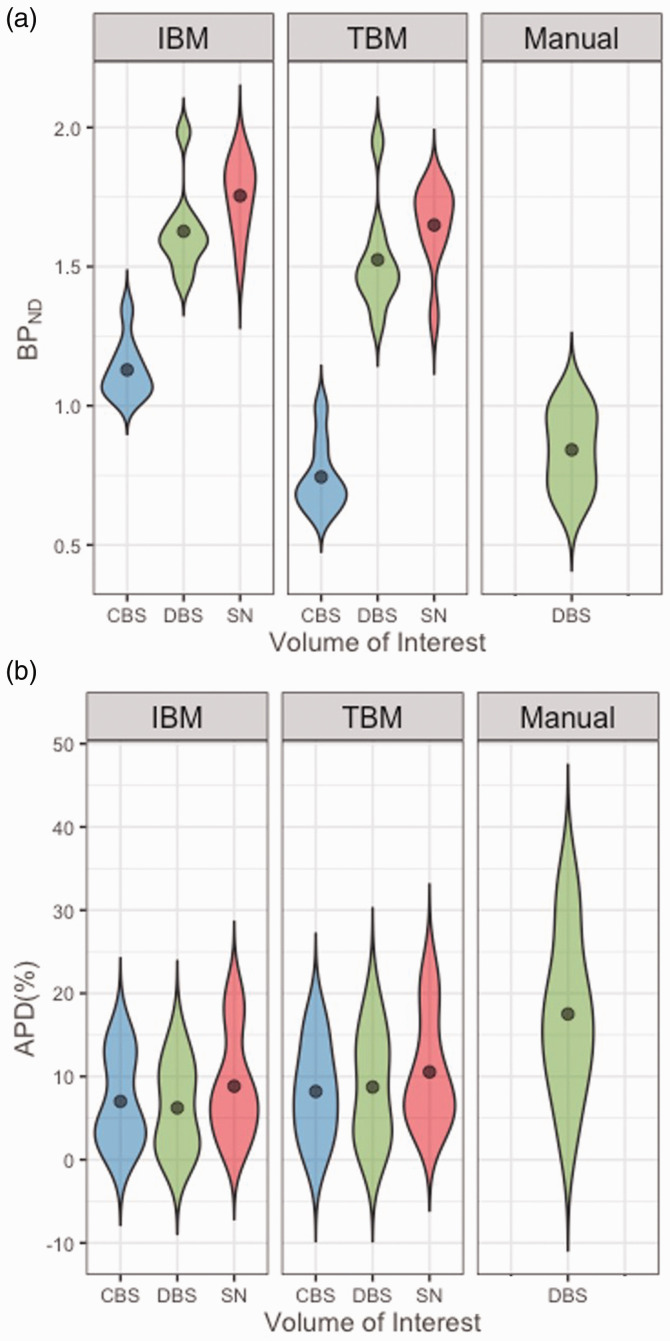
Violin plots of A: BP_ND_ values, averaged per subject, and B: Absolute Percentage Difference (APD) in the different delineation methods. CBS: caudal brainstem; DBS: dorsal brainstem; IBM: individual-based method; SN: substantia nigra; TBM: template-based method.

Test-retest reliability was assessed for using the different PET VOI-delineation methods for calculation of the regional BP_ND_ values (Figure 2(a), Table S3). Outcome values for APD, ICC, SEM and MD are displayed in Table 2 and Figure 2(b). Both automatic brainstem VOI defining methods, based on individual or on template data, showed improvement in APD, SEM and MD compared to the conventional method (Table S4). Both semi-automatic methods provided BP_ND_ values that were consistently higher and presented a consistently lower COV than those obtained with the conventional method. This effect was strongest in the individual-based method for all VOIs. For the dorsal brainstem VOI, the individual-based method showed highest differentiability (ICC), lowest intra-subject differences (APD), smallest measurement error (SEM) and lowest MD. In the case of the substantia nigra VOI, the individual-based method also showed lowest APD, SEM and MD, but ICC was higher for the template-based method. For the caudal brainstem VOI, the template-based method showed highest ICC and lowest SEM and MD, but similar APD compared to the one using the individual-based method.

**Table 2.** Test-retest metrics for BP_ND_ value quantification using the template-based method and the individual-based method.

Volume of interest	Template-based method				Individual-based method			
	Avg APD (%)	ICC	SEM	MD (%)	Avg APD (%)	ICC	SEM	MD (%)
Dorsal brainstem	8.73	0.72	0.11	19.86	6.23	0.74	0.09	14.76
Substantia nigra	10.55	0.39	0.13	21.69	8.83	0.32	0.12	18.64
Caudal brainstem	8.2	0.84	0.05	19.78	7.01	0.65	0.07	16.93

avg APD: average absolute percentage difference; DBS: dorsal brainstem; ICC: intraclass correlation coefficient; SEM: standard error of measurement; MD: minimal detectable difference.

**Figure 3.**
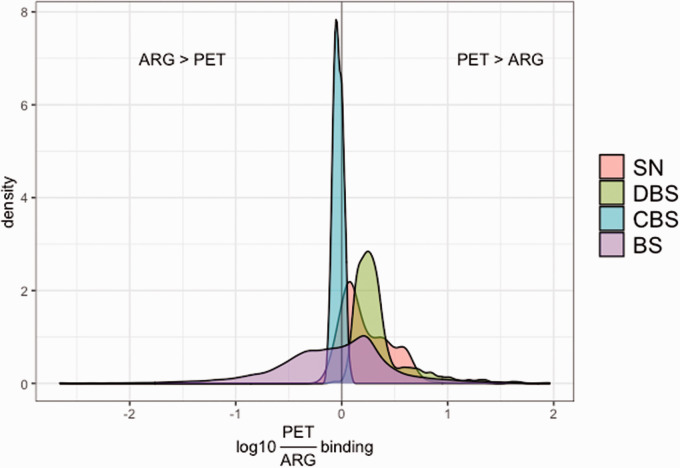
Density plot of the log10 ratio of [^11^C]AZ10419369 PET BP_ND_ over [^3^H]AZ10419369 3 D ARG specific binding in each voxel within the different analyzed volumes of interest: substantia nigra (SN), dorsal brainstem (DBS), caudal brainstem (CBS) and rest of the brainstem (BS).

Comparison of the PET template data and the 3D-ARG data showed that throughout most of the brainstem, normalized BP_ND_ values were relatively higher than normalized specific binding values, particularly in the dorsal brainstem VOIs (Figure 3). In most of the voxels in the caudal brainstem VOI, normalized specific binding values were relatively higher.

## Discussion

In the present study, we present an improved method for VOI delineation of [^11^C]AZ10419369 PET data in the brainstem compared to a previously applied method.^14^ We generated three suitable VOIs for analysis of [^11^C]AZ10419369 binding, with the help of a high-resolution 3D model of [^3^H]AZ10419369 binding generated from ARG images and compared two semi-automatic VOI delineation methods. We observed better reliability (APD), smaller measurement error (SEM) and were able to detect proportionally smaller differences (MD) for both methods compared to a conventional manual method when analyzing [^11^C]AZ10419369 binding in the brainstem. Although differentiability (ICC) was relatively high for the manual method, especially for the substantia nigra VOI, variance within and between subjects was much higher than for the semi-automatic methods. The manual method results in much larger minimal detectable differences and can therefore be considered an inferior method. Good reliability outcomes were seen for the individual-based method, although VOIs based on individual data have to be generated for every new subject and could therefore be more error-prone. Moreover, an advantage of the template-based method is its easy implementation for future [^11^C]AZ10419369 PET data, which encourages widespread use and therefore improves generalizability of outcome values. In this study, the template VOIs were tested on a separate dataset from which they were created, in order to provide a better estimation of the reliability for future use. It should be noted that solely data of healthy subjects was used. Therefore, future studies should confirm the reliability in subjects with psychiatric or neurological disorders.

Both methods used structural information provided by literature to determine VOI sizes, which can be seen as a limitation to these methods. The size of VOIs created using the template-based method were further dependent on structural differences between template and individual space and therefore shows more flexibility in VOI sizes (Table S1).

### Dorsal brainstem

The individual-based method showed highest improvement of test-retest metric outcomes for the dorsal brainstem VOI, with higher differentiability (ICC), lower intra-subject differences (APD), slightly higher precision of individual scores (SEM) and a lower MD. The template-based method might account less for inter-subject variability of 5-HT_1B_ receptor distributions in the dorsal brainstem.

Based on the continuous, rather than discrete, [^3^H]AZ10419369 binding seen in the 3D ARG model, we chose to combine discrete anatomical volumes in this VOI. Localization of these anatomical volumes in the final template dorsal brainstem VOI was confirmed for the dorsal raphe nucleus, periaqueductal gray and the superior part of the median raphe nucleus by overlaying the masks of the AAN atlas.^59^ Visual inspection on the MRI template also confirmed the localization of a large part, medially located, of the inferior and superior colliculi in the final template VOI. Analysis of the data in separate VOIs for the dorsal and median raphe nuclei, periaqueductal gray, superior and inferior nuclei, generated lower reliability (Table S5) than when using a combined VOI (Table S4). Due to the continuity of the signal, these VOIs were prone to overlapping each other, which could likely explain the resulting lower reliability.

### Substantia nigra

For the substantia nigra VOI, the individual-based method resulted in better reliability outcomes, except for the differentiability of average BP_ND_ values in substantia nigra, which was higher when using the template-based method. Although the substantia nigra was shown to be a region with relatively high 5-HT_1B_ receptor densities, differentiability between subjects was shown to be low (ICC: 0.32 and 0.39 for individual-based and template-based method respectively). This can be explained by the combination of a slightly larger difference between PET1 and PET2 (APD, Table 2), and the relatively smaller difference between subjects (COV, Table S3). The relatively homogeneous testing group with males of similar age could explain similar outcome values between subjects. Although in the template data no significant correlations were found between BP_ND_ in this VOI and age of the subject, female subjects had significantly higher BP_ND_ values than male subjects (mean ± SD: 1.96 ± 0.32 vs 1.64 ± 0.30, p < 0.001). It should be further studied if a better reliability can be shown with a more heterogeneous testing group, to see if data-driven approaches can also be suitable for reliable analysis of [^11^C]AZ10419369 BP_ND_ in the substantia nigra.

Localization of the substantia nigra within the final template VOI was confirmed by overlaying the masks of the ATAG template to the PET data.^34^

### Caudal brainstem

The template-based method provided better differentiability, precision and lower MD for the caudal brainstem VOI, but slightly higher intra-subject differences compared to the individual-based method. As this VOI has substantially lower BP_ND_ (Table S3) and lower volume (Table S1) than the two other analyzed VOIs, this implicates that the template-based method gives an advantage in small regions with lower receptor densities. The lower signal-to-noise ratio may lead to more random VOI-selection when based on individual data. It should be noted that the high ICC for BP_ND_ in this VOI seems to be mostly driven by a higher variance between subjects (Coefficient of Variation, Table S3), while differences between the outcomes of the two PET examinations are similar to those found for the other VOIs. Future studies should confirm if the inter-subject variability is a result of variability in individual 5-HT_1B_ densities in this region rather than a result of the VOI-delineation method.

In order to choose the volume of the caudal brainstem VOI, we used a data-driven approach in which the reliability outcomes and BP_ND_ values were taken into account. Although the limited literature on known 5-HT_1B_ receptor densities in the caudal part of the human brainstem only reports on substantial receptor binding in the medulla,^23^ in rodents 5-HT_1B_ receptor densities have been found in the pons as well.^24^ Both our ARG and PET data showed AZ10419369 binding in the pons. Therefore, we included the caudal part of the pons as well to set the liberal VOI before erosion towards the final VOI. Both the VOIs resulting from the individual-based method and the template-based VOI were located in the caudal part of the pons and dorsal part of the medulla oblongata.

### ARG vs PET

In this study, findings based on *post mortem* ARG data were used to study 5-HT_1B_ receptor distribution at high resolution. As anatomy of the studied subjects in the ARG experiments might differ from the subjects who underwent PET examinations, differences in 5-HT_1B_ receptor distribution might occur. Moreover, factors such as receptor degradation due to a *post mortem* interval could influence receptor binding in ARG. *In vivo* studies can be elsewise affected, such as by binding of (fluctuating levels of) endogenous compounds. Thus, to evaluate to what extent receptor distribution measured with ARG can be used to guide VOI definition in *in vivo* receptor quantification with PET, we also investigated the relationship between *post mortem* [^3^H]AZ10419369 specific binding in whole hemisphere ARG data of three subjects and BP_ND_ in the reported [^11^C]AZ10419369 test retest PET data (see Supplementary section 1.3 and 2.2). Binding was measured in nine VOIs outside the brainstem and showed a strong, significant correlation (r = 0.78, p = 0.013).

The comparison of PET template data voxel-by-voxel with the 3D ARG data showed that normalized BP_ND_ values per voxel were generally higher than normalized specific binding values. This is represented by a general shift towards the right of the density plots displayed in Figure 3. This phenomenon can be explained by the normalization method: high receptor densities measured in PET are more affected by spill-out of signal, resulting in underestimation of the highest binding value towards which normalization is carried out. The substantia nigra VOI displayed a relatively good agreement between the two modalities and less overestimation of BP_ND_ versus specific binding values. An overestimation of PET BP_ND_’s compared to ARG specific binding was observed in the dorsal brainstem. This VOI might be affected in PET by spill-in from nearby high [^11^C]AZ10419369 binding regions such as the substantia nigra. It should be noted that small deformations present in the ARG slides could have locally introduced underestimated values, affecting the ARG binding negatively in the dorsal brainstem region and to a lesser extent the substantia nigra. The caudal brainstem showed a slight underestimation of BP_ND_ values compared to specific binding values. As this region has relatively lower binding values, it is more affected by the lower sensitivity of PET. Improved resolution in future PET systems can possibly alter regional BP_ND_ values in the brainstem, particularly in the dorsal brainstem. In the PET data, an effect of age on BP_ND_ values was found in the dorsal and caudal brainstem. It should be noted that the ARG data here is based on one subject, which differed in age compared to the studied subjects for the PET test-retest data. Therefore, the ARG data in the dorsal and caudal brainstem was adjusted for age.

## Conclusion

In conclusion, using the provided VOIs for brainstem regions, both the template-based method and the individual based method showed an improvement in reliability for 5-HT_1B_ receptor quantification in the brainstem compared to conventional methods. Both the dorsal brainstem and caudal brainstem VOI could be quantified with high reliability in healthy subjects, however quantification in the substantia nigra remains suboptimal with the tested approaches. The template VOIs can easily be implemented in future analyses of 5-HT_1B_ receptor binding in the brainstem. Application of the template VOIs in data of subjects with psychiatric or neurological disorders has yet to be validated.

## Supplementary Material

Supplementary material

Supplementary material
