# Supplementary material for: Serotonin 1B receptor density mapping of the human brainstem using positron emission tomography and autoradiography
Source: J Cereb Blood Flow Metab. 2021 Oct 13;42(4):630–41. doi: 10.1177/0271678X211049185 (PMC8943614; doi:10.1177/0271678X211049185)
Supplement: Supplementary material [file sj-pdf-1-jcb-10.1177_0271678X211049185.pdf]

Supplementary section

1. Supplementary Material and methods

1.1 Autoradiography

1.1.1 [^{3}H]AZ10419369 in vitro autoradiography

Sections were incubated at room temperature for 60 minutes in 1.5 nM [^{3}H]AZ10419369[1] in buffer containing Tris-HCl 50 mM, pH 7.4 incl. 4 mM MgCl, 4 mM $CaCl_2$. 0.1% BSA was added to the incubation buffer to decrease non-specific binding, e.g. to the tissue embedding material carboxymethyl cellulose. Adjacent sections were simultaneously incubated in the previously mentioned buffer with addition of 10 µM 5-HT. After washing and drying, sections were placed together with autoradiographic micro-scale standards (American Radiolabeled Chemicals Inc.), and exposed to phosphor imaging plates (Fujifilm Plate BAS-TR2025, Fujifilm, Tokyo, Japan) for four days. Image radioactivity was detected using a Fujifilm BAS-5000 phosphor imager (Fujifilm, Tokyo, Japan), which resulted in scanned images with signal representing average photostimulated luminescence (PSL)/mm^2.

1.1.2 Nissl staining

Sections used for autoradiography were subsequently used for Nissl staining. In brief, slides were stained with cresyl violet (Histolab, Göteborg, Sweden), dehydrated in consecutively in increasing concentrations of ethanol, immersed in Histolab Clear (xylene substitution, Histolab, Göteborg, Sweden), dried and mounted. Each Nissl-stained section was digitized using an Epson Perfection V800 Photo scanner (Seiko Epson, Tokyo, Japan).

1.1.3 Defining regions of interest

On each scanned section regions of interest (ROIs) were manually drawn using Multi Gauge 3.2 phosphor imager software (Fujifilm, Tokyo, Japan). Using the processed micro-scale standards, mean photostimulated luminescence (PSL/mm^2) of each ROI was transformed into radioactivity values and binding density (pmol/mg tissue wet weight). Specific binding was determined by subtracting the level of non-specific binding from the total binding.

1.2 PET VOI-delineation methods in the brainstem

Table S1: Sizes for VOIs using the template-based and the individual-based method

Volume of interest	template-based method	individual-based method	Reference
	avg volume ± SD	volume	
**Dorsal Brainstem**	1123.16 ± 100.99	1100	PAG: Chen et al., 2017<br>DRN/MRN: Kranz et al., 2012<br>SC/IC: Cecchetti et al., 2016
**Substantia Nigra**	965.44 ± 82.41	1000	Murty et al., 2014
**Caudal Brainstem**	277.31 ± 42.04	250	(method) Schain et al., 2013

1.3 Whole Brain ARG and PET comparison

The correlation between 5-HT_{1B} receptor binding measured *in vivo* with PET and *in vitro* using autoradiography was studied. ROIs outside of the brainstem were chosen to include a broad range of 5-HT_{1B} receptor binding densities, based on previous findings in the literature.[7,8]

1.3.1 Autoradiography

Necessary materials were as described in the main paper (see 2.1.1).

1.3.1.1 Human postmortem brain tissue

Human brains of three donors (see Table S2) were obtained postmortem at clinical autopsy at the National Institute of Forensic Medicine, Karolinska Institutet (Stockholm, Sweden) and at the Human Brain Tissue Bank (Budapest, Hungary).
Whole hemispheres were removed, frozen, and cryosectioned as described previously.[9] A heavy-duty cryomicrotome (Leica cryomacrocut CM3600, Leica, Nussloch, Germany) was used to cut whole hemispheres into 100µ-thick horizontal cryosections. Subsequently, cryosections were transferred to gelatinized or poly-l-lysine-treated glass plates (10 x 22 cm), dried at room temperature and stored with dehydrating agents at -25 ºC until use.

Table S2: Specifications of used brain tissue

Brain no.	Level (z-direction)	Age (y)	Sex	PMI (h)	Cause of death
**1**	1-3	54	M	22-23	Coronary heart disease
**2**	1&3	59	F	11	Cardiac failure in septic state
**3**	2-3	63	m	48	Myocardial infarction

PMI: Post Mortem Interval

1.3.1.2 [^{3}H]AZ10419369 in vitro autoradiography

The autoradiographic procedure and delineation of ROIs were as described for the ARG procedure in the brainstem (see S1.1). The nissl staining procedure was optimized for whole hemisphere tissue. Regional specific binding values were averaged within subjects and subsequently between subjects.

1.3.2 Positron Emission Tomography

PET and MRI imaging analyses was performed as reported for creation of the brainstem VOIs in the main manuscript. Data of the 8 test subjects was used, regional BP_{ND} values of the test and retest PET occasion were averaged.

The FSL Harvard-Oxford subcortical and MNI structural atlas[10] were used for automatic delineation of subcortical and cortical ROIs, respectively. ROIs were thresholded at 25% when transforming to individual space. The following ROIs were included in the analysis: thalamus, hippocampus, caudate nucleus, putamen, ventral striatum, frontal cortex, anterior cingulate cortex, insular cortex and occipital cortex.

1.3.2.2 Statistical analysis

To assess the correlation between average regional BP_{ND} values measured with PET and specific binding measured with whole hemisphere ARG, a Pearson correlation analysis was used.

2. Supplementary Results

2.1 Comparison of PET VOI-defining methods

Table S3: Positron Emission Tomography BP_{ND} and Autoradiography specific binding

Volume/Region of interest	PET TBM		PET IBM		ARG specific binding (pmol/g) ± SD
	BP_{ND} ± SD	COV	BP_{ND} ± SD	COV	
**Dorsal Brainstem**	1.52 ± 0.21	0.14	1.63 ± 0.17	0.10	69.77 ± 18.09
**Substantia Nigra**	1.65 ± 0.17	0.10	1.75 ± 0.14	0.08	159.62 ± 20.07
**Caudal Brainstem**	0.74 ± 0.13	0.18	1.13 ± 0.12	0.10	15.82 ± 7.92

COV: Coefficient of Variation (between subject SD/mean); DBS: dorsal brainstem; IBM: individual-based method; TBM: template-based method

Table S4: Test-retest metrics for BP_{ND} value quantification using the manual VOI defining method

Volume of interest	BP_{ND} ± SD	COV	avg APD ± SD (%)	ICC	SEM	MD (%)
**Dorsal Brainstem**	0.79 ± 0.38	48.23	35.14 ± 29.19	0.69	0.21	24.95
**Substantia Nigra**	1.01 ± 0.28	27.55	20.12 ± 13.7	0.69	0.16	58.21
**Caudal Brainstem**	0.27 ± 0.2	75.63	31.98 ± 29.53	0.82	0.09	44.35

Table S5: Test-retest metrics for BP_{ND} value quantification using the template-based method and the individual-based method, dorsal brainstem VOI as separate VOIs.

Volume of interest	template-based method				individual-based method			
	avg APD ± SD (%)	ICC	SEM	MD (%)	avg APD ± SD (%)	ICC	SEM	MD (%)
**Dorsal RN**	13.92 ± 11.07	0.64	0.17	27.36	11.04 ± 6.97	0.64	0.17	24.71
**PAG**	9.85 ± 7.82	0.57	0.16	24.82	7.89 ± 5.94	0.58	0.13	18.67
**Median RN**	12.79 ± 9.34	0.58	0.13	28.29	6.94 ± 5.07	0.73	0.10	16.28
**Superior Colliculi**	14.40 ± 10.98	0.57	0.14	34.13	13.39 ± 9.56	0.58	0.13	33.05
**Inferior Colliculi**	14.25 ± 9.45	0.61	0.16	30.45	14.52 ± 12.38	0.44	0.18	32.97

avg APD: average absolute percentage difference; ICC: intraclass correlation coefficient; PAG: periaqueductal gray; RN: Raphe Nucleus; SEM: standard error of measurement; MD: Minimal Detectable difference

2.2 Whole Brain ARG and PET comparison

BP_{ND} values of the ROIs on [^{11}C]AZ10419369 parametric images of 8 subjects correlated strongly with specific binding values measured by whole hemisphere ARG (r=0.78, p=0.013, rho=0.77). The correlation can be seen Figure S1, in which values of each modality are normalized to the binding values of the nucleus accumbens. Therefore, a slope of 1 would represent absolute correlation. The line of best fit has a slope of 0.85 and an intercept of -5.99%, indicating that a region lacking specific binding measured in ARG would result in a BP_{ND} > 0 when measured with PET. See Figure S2 for representative autoradiograms.

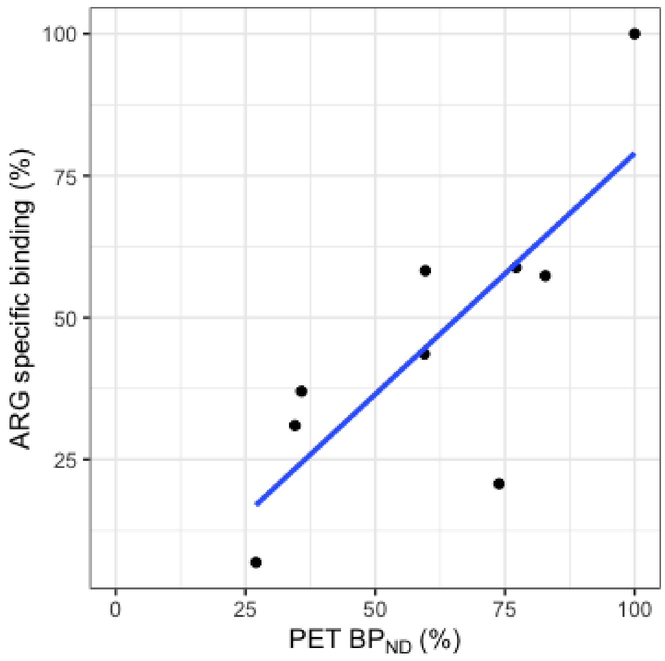

Figure S1: Correlation between [^{11}C]AZ10419369 BP_{ND} as measured with PET and binding densities measured with [^{3}H]AZ10419369 whole hemisphere autoradiography. p=0.013 r=0.78

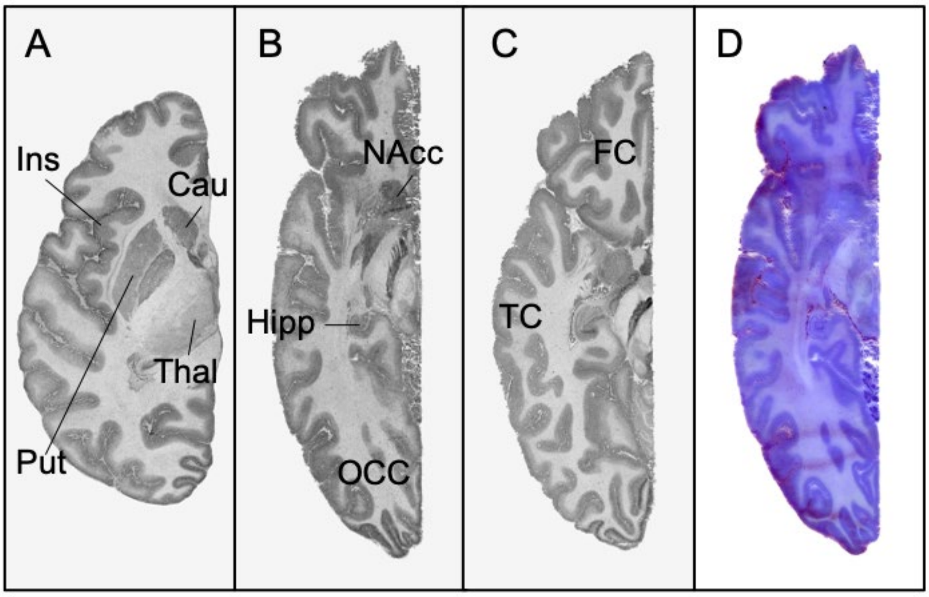

Figure S2: [^{3}H]AZ10419369 ARG binding distribution on whole brain hemispheres, examples. A: Subject1, level 1; B: Subject 2, level 2; C: Subject 3, level 3. D: Cresyl violet staining (subject 2, level 2). Cau: Caudate nucleus; FC: Frontal cortex; Hipp: Hippocampus; Ins: Insular cortex; MRN: NAcc: Nucleus accumbens; OCC: Occipital cortex; Put: Putamen; Thal: Thalamus; TC: Temporal cortex.

Supplementary legends

Video 1: Pattern of distribution of [^{3}H]AZ10419369 ARG specific binding in the brainstem. The animation is created using ParaView.[11]

Supplementary References

1. Pierson ME, Andersson J, Nyberg S, et al. [11C]AZ10419369: A selective 5-HT1B receptor radioligand suitable for positron emission tomography (PET). Characterization in the primate brain. *Neuroimage* 2008; 41: 1075–1085.
2. Chen Z, Chen X, Liu M, et al. Volume gain of periaqueductal gray in medication-overuse headache. *J Headache Pain*; 18. Epub ahead of print 2017. DOI: 10.1186/s10194-016-0715-9.
3. Kranz GS, Hahn A, Savli M, et al. Challenges in the differentiation of midbrain raphe nuclei in neuroimaging research. *Proc Natl Acad Sci U S A* 2012; 109: E2000–E2000.
4. Cecchetti L, Ricciardi E, Handjaras G, et al. Congenital blindness affects diencephalic but not mesencephalic structures in the human brain. *Brain Struct Funct* 2016; 221: 1465–1480.
5. Murty VP, Shermohammed M, Smith D V., et al. Resting state networks distinguish human ventral tegmental area from substantia nigra. *Neuroimage* 2014; 100: 580–589.
6. Schain M, Tóth M, Cselényi Z, et al. Improved mapping and quantification of serotonin transporter availability in the human brainstem with the HRRT. *Eur J Nucl Med Mol Imaging* 2013; 40: 228–237.
7. Varnäs K, Nyberg S, Halldin C, et al. Quantitative analysis of [11C]AZ10419369 binding to 5-HT1B receptors in human brain. *J Cereb Blood Flow Metab* 2011; 31: 113–123.
8. Varnäs K, Hall H, Bonaventure P, et al. Autoradiographic mapping of 5-HT1B and 5-HT1D receptors in the post mortem human brain using [3H]GR 125743.

Brain Res 2001; 915: 47–57.

9. Hall H, Halldin C, Farde L, et al. Whole hemisphere autoradiography of the postmortem human brain11Proceedings from COST meeting, “Preclinical Pharmacological Studies with and for Radiopharmaceuticals,” held at Orsay, France, October 24–25, 1996. *Nucl Med Biol* 1998; 25: 715–719.
10. Diedrichsen J, Balsters JH, Flavell J, et al. A probabilistic MR atlas of the human cerebellum. *Neuroimage* 2009; 46: 39–46.
11. Ahrens J, Geveci B, Law C. ParaView: An end-user tool for large-data visualization. In: *Visualization Handbook*. 2005. DOI: 10.1016/B978-012387582-2/50038-1.
